# CB2 Receptor as Emerging Anti-Inflammatory Target in Duchenne Muscular Dystrophy

**DOI:** 10.3390/ijms24043345

**Published:** 2023-02-07

**Authors:** Maura Argenziano, Vincenzo Pota, Alessandra Di Paola, Chiara Tortora, Maria Maddalena Marrapodi, Giulia Giliberti, Domenico Roberti, Maria Caterina Pace, Francesca Rossi

**Affiliations:** 1 Department of Woman, Child and General and Specialistic Surgery, University of Campania “Luigi Vanvitelli”, Via Luigi De Crecchio, 80138 Naples, Italy; 2 Centro Clinico NeMO, Via Leonardo Bianchi, 80131 Naples, Italy; 3 Department of Experimental Medicine, University of Campania “Luigi Vanvitelli”, Via Luigi De Crecchio, 80138 Naples, Italy

**Keywords:** Duchenne muscular dystrophy, macrophage phenotype, inflammation, CB2 receptor

## Abstract

Duchenne Muscular Dystrophy (DMD) is a very severe X-linked dystrophinopathy. It is due to a mutation in the DMD gene and causes muscular degeneration in conjunction with several secondary co-morbidities, such cardiomyopathy and respiratory failure. DMD is characterized by a chronic inflammatory state, and corticosteroids represent the main therapy for these patients. To contradict drug-related side effects, there is need for novel and more safe therapeutic strategies. Macrophages are immune cells stringently involved in both physiological and pathological inflammatory processes. They express the CB2 receptor, one of the main elements of the endocannabinoid system, and have been proposed as an anti-inflammatory target in several inflammatory and immune diseases. We observed a lower expression of the CB2 receptor in DMD-associated macrophages, hypothesizing its involvement in the pathogenesis of this pathology. Therefore, we analyzed the effect of JWH-133, a CB2 receptor selective agonist, on DMD-associated primary macrophages. Our study describes the beneficial effect of JWH-133 in counteracting inflammation by inhibiting pro-inflammatory cytokines release and by directing macrophages’ phenotype toward the M2 anti-inflammatory one.

## 1. Introduction

Duchenne muscular dystrophy (DMD), a severe and progressive dystrophinopathy, affects early childhood and is mainly characterized by muscle wasting, myonecrosis, and chronic inflammation [1,2]. It is an X-linked disease chiefly due to a deficiency of dystrophin protein—encoded by DMD gene—with an incidence of 1 in 3500 boys all over the world [3]. Dystrophin protein is a key element of sarcolemma; it creates an essential linkage between the muscle cell cytoskeleton and the extracellular matrix, interacting with several glycoproteins in the cell membrane (i.e., sarcoglycans, dystrobrevins, dystrobrevins, etc.) [4] and maintaining muscular structure integrity [5]. Any mutation in the DMD gene leads to dysfunction of the dystrophin protein and consequently all dystrophin-associated glycoproteinsm, which furthermore causes muscular degeneration such as DMD and the more benign Becker type of dystrophinopathy [6,7]. DMD can be better described as a multi-systemic disease (5), since, besides musculature, it also affects many other organs and districts such as the heart, lungs, nervous system, and liver [8]. Cardiomyopathy and respiratory failure are the most frequent co-morbidities in DMD patients, thus making necessary treatments with cardiac drugs and ventilatory assistance [9]. In the last two decades, several authors delineated the critical role of persistent chronic inflammation in the pathogenesis of DMD [10,11]. It is mainly caused by dystrophin-deficient myofibers damage and the consequent cycles of their necrosis and regeneration [12]. Inflammation is normally fundamental for tissue restoration after damage; nevertheless, when it becomes chronic, it triggers a vicious circle in which myofiber damage-related inflammation stimulates the production of further pro-inflammatory cytokines as well as the immune cells infiltration in muscle. These events exacerbate the pro-inflammatory balance, causing fibrosis and stiffness in muscular tissues [13]. Since recent years, the role of resident macrophages in modulating muscle tissue metabolism emerged; the inhibition of their differentiation, indeed, has a protective role in the mouse model of DMD [14]. Macrophages are phagocytic mononuclear cells with a key role in both inflammatory processes and immune response. They can be present in two characterized and archetypal different phenotypes: “M1-macrophages”, or classically activated macrophages with pro-inflammatory functions, and “M2-macrophages”, or alternatively activated macrophages exerting anti-inflammatory and immunosuppressive activities [15]. The imbalance in M1/M2 ratio with a M1 prevalence can be observed in several inflammatory and autoimmune diseases [16,17,18], which thus permits to hypothesize their involvement in the pathogenesis of these diseases. Macrophages are known to express cannabinoid receptor 2 (CB2), as well as all the other peripheral cells involved in immune response [19]. CB2 is one of the main elements of endocannabinoid system (ECS), a neurotransmitter system with an important role in several physiological and pathological processes, namely embryonic development, inflammation, immune response, cancer, and infection [20]. For these reasons, as well considering the protective effects observed after the proper stimulation of CB2 receptor, ECS has been proposed as a novel target for cancer, autoimmune, and inflammatory diseases. [18,20,21,22,23,24,25,26,27,28]. Iron metabolism alteration delineated the inflammatory status; in particular, a high concentration of pro-inflammatory cytokines (such IL-6) could cause an increase in hepcidin levels that is responsible for intracellular iron accumulation, since it induces the degradation of ferroportin-1 (FPN-1). FPN-1 is the only known iron exporter [29], whereas transferrin receptor 1 (TfR1) is one of the iron exporters located on cell membranes and is able to internalize the iron-transferrin complex. Iron metabolism is finely regulated in several physiological processes, and its alteration as in inflammation could actively participate to pathology onset [30]. Indeed, the use of iron chelators in clinical practice is prevalent, especially in hematological pathologies [31], and exhibits a promising proposal for other kind of diseases [17,32,33,34,35].

DMD therapy protocol mainly consists in the administration of corticosteroids because of their anti-inflammatory functions [36,37,38,39,40] and the consequent capability to prolong patients’ ambulation. This therapeutic regimen maintains muscle strength and extends life expectancy, but corticosteroids long-term use could cause severe and very debilitating side effects (i.e., weight gain, glucose intolerance, hypertension, and depression) [41,42], thus making necessary the identification of an alternative and less toxic approach. Recently, the most intriguing therapeutical proposals are represented by stop-codon read-through therapy, gene replacement approaches, myoblast transfer therapy, and also drug-related inhibition of resident macrophages [43,44,45]. We suggest CB2 receptor agonism as an alternative, effective, and safe anti-inflammatory target for the therapy of DMD.

## 2. Results

### 2.1. Evaluation of Inflammatory State: Pro- and Anti-Inflammatory Cytokines Release and Iron Internalization

**Figure 1:**
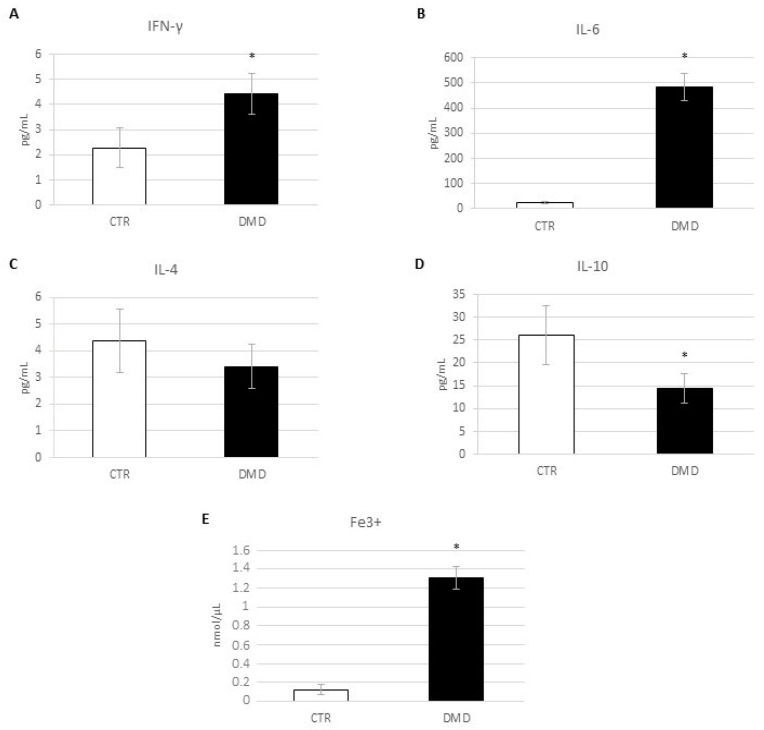
Levels of IFN-γ (**A**), IL-6 (**B**), IL-4 (**C**), and IL-10 (**D**) from control macrophages (CTR) and Duchenne muscular dystrophy macrophages (DMD) investigated through an enzyme-linked immunosorbent assay (ELISA). The graphs show interleukins levels [pg/mL] as the mean ± standard deviation (SD). (**B**) Fe^3+^ intracellular concentrations (nmol/µL) in CTR and DMD-associated macrophages, determined by iron assay. Histogram shows Fe^3+^ concentration as the mean ± SD. Students’ *t*-test has been used for statistical analysis. *, *p* ≤ 0.05 compared to CTR (*p*-values: (**A**) 0.0457; (**B**) 0.0066; (**D**) 0.0417; (**E**) 0.0098).

Figure 1 depicts the graphical representation of the inflammatory state of DMD-associated macrophages. We analyzed the released levels of INF-γ and IL-6 (Figure 1A,B), as pro-inflammatory cytokines and of IL-10 and IL-4 (Figure 1C,D) as anti-inflammatory cytokines using the ELISA test. Although we observed very high levels of pro-inflammatory molecules from DMD patients’ macrophages, the levels of anti-inflammatory cytokines appeared lower in DMD-associated than in healthy macrophages (CTR). By means of an Iron Assay experiment, we also measured the intracellular levels of iron, being that the intracellular accumulation of this element is a key indicator of an inflammatory state. Moreover, we observed a clear signal related to a pro-inflammatory condition: a hyper-internalization of iron in macrophages from DMD patients, in comparison with healthy macrophages (almost 0.18 nmol/µL in DMD versus 0.01 nmol/µLin CTR) (Figure 1E).

### 2.2. Macrophage Phenotype Characterization

**Figure 2:**
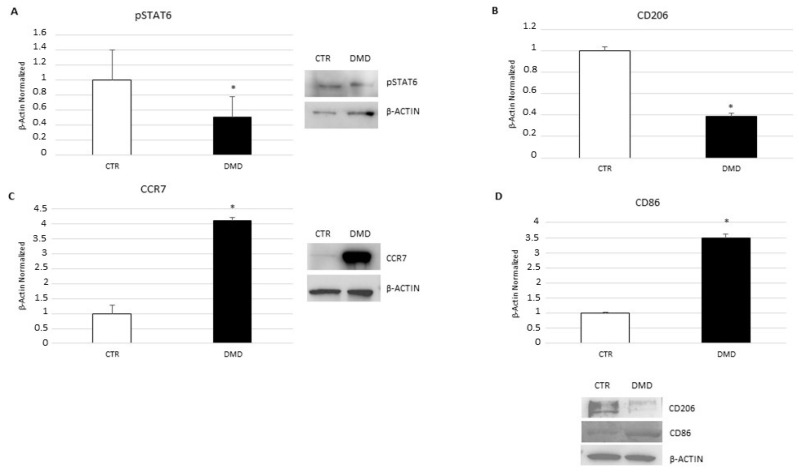
pSTAT6 (**A**), CD206 (**B**), CCR7 (**C**), and CD86 (**D**) protein expression levels in CTR macrophages and DMD-associated macrophages evaluated by Western blot technique, starting from 15 μg of total lysates. The most representative images are displayed. The protein bands were detected through Image Lab. Ink software “BIORAD”, and the intensity ratios of immunoblots compared to CTR, taken as 1, were quantified after normalizing with respective controls. The relative quantification for these proteins, normalized for the housekeeping protein β-actin, is represented in the histogram as the mean ± SD. Students’ *t*-test has been used for statistical analysis. *, *p* ≤ 0.05 compared to NT (*p*-values: (**A**) 0.0003; (**B**) 0.0012; (**C**) 0.0069; (**D**) 0.0521).

By performing Western blotting, we evaluated the expression of pSTAT6 (Figure 2A) and CD206 (Figure 2B) proteins, which areM2 macrophage phenotype markers, and also of CCR7 (Figure 2C) and CD86 (Figure 2D) proteins, as markers of M1 macrophage phenotype. In DMD-associated macrophages, both M2 markers are strongly down-expressed, whereas the M1 markers’ expression levels are higher in comparison with CTR macrophages.

### 2.3. CB2 Receptor Expression Level in DMD-Associated Macrophages

**Figure 3:**
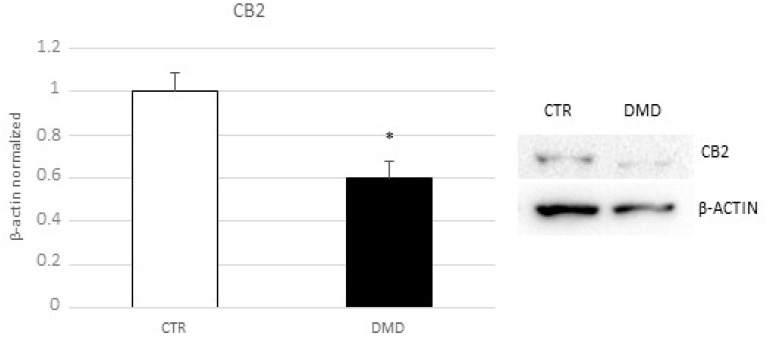
CB2 receptor protein expression levels in CTR and DMD-associated macrophages, determined by Western blot, starting from 15 μg of total lysates. The most representative images are displayed. The protein bands were detected through Image Lab. Ink software “BIORAD”, and the intensity ratios of immunoblots compared to CTR, taken as 1, were quantified after normalizing with respective controls. The relative quantification for CB2 receptor expression, normalized for the housekeeping protein β-actin, is represented in the histogram as the mean ± SD. Students’ *t*-test has been used for statistical analysis. *, *p* ≤ 0.05 compared to CTR (*p*-value: 0.0249).

By Western blotting, we observed a statistically significant reduction of CB2 receptor protein expression level in macrophages from DMD patients compared with macrophages from healthy donors (Figure 3). CB2 receptor is normally expressed in immune cells, including macrophages, and it is co-responsible for their specific functions.

### 2.4. Effect of CB2 Receptor Stimulation on Inflammatory State

**Figure 4:**
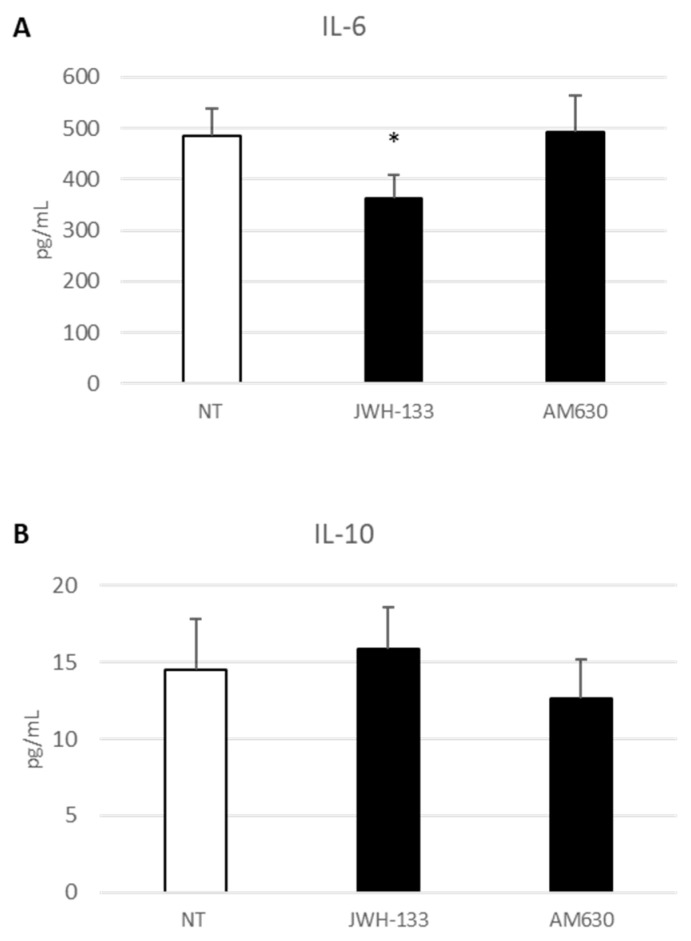
Levels of IL-6 (**A**) and IL-10 (**B**) from DMD-associated macrophages after 24-h treatment with JWH-133 and AM630, investigated through enzyme-linked immunosorbent assay (ELISA). The graphs show interleukins levels [pg/mL] as the mean ± standard deviation (SD). Students’ *t*-test has been used for statistical analysis. *, *p* ≤ 0.05 compared to NT (*p*-value: (**A**) 0.0350).

**Figure 5:**
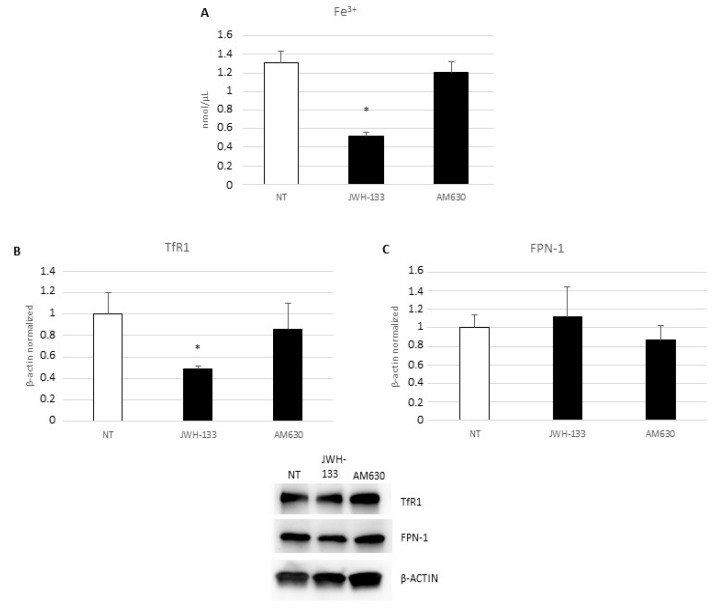
(**A**) Fe^3+^ intracellular concentrations (nmol/µL) in DMD-associated macrophages after 24-h treatment with JWH-133 and AM630, determined by iron assay. Histogram shows Fe^3+^ concentration as the mean ± SD. TfR1 (**B**) and FPN-1 (**C**) protein expression levels in DMD-associated macrophages after 24-h treatment with JWH-133 and AM630, determined by Western blotting, starting from 15 μg of total lysates. The most representative images are displayed. The protein bands were detected through Image Lab. Ink software “BIORAD”, and the intensity ratios of immunoblots compared to non-treated (NT), taken as 1, were quantified after normalizing with respective controls. The relative quantification for TfR1 and FPN-1 expression, normalized for the housekeeping protein β-actin, is represented in the histogram as the mean ± SD. Students’ *t*-test has been used for statistical analysis. *, *p* ≤ 0.05 compared to NT (*p*-values: (**A**) 0.0169; (**B**) 0.0509).

The ELISA revealed a rebalance of cytokines, released after treatment of DMD-associated macrophages with JWH-133 and AM630, in favor of an anti-inflammatory profile. There is a significant decrease in IL-6 production (Figure 4A) and an evident trend in the increase of the production of IL-10 (Figure 4B) pro- and anti-inflammatory molecules, respectively. Remaining in the field of inflammatory state evaluation, we also studied iron metabolism. With an iron assay, we evidenced a statistically significant reduction of intracellular levels of iron in macrophages treated with JWH-133 (Figure 5A). With Western blotting, we studied the effect on the two main iron transporters: TfR1, which is normally responsible for iron internalization in cell cytoplasm, and FPN, the only known iron exporter located on cell surface. JWH-133 exposure causes a significant reduction in TfR1 protein expression level (Figure 5B) and a trend to increase in FPN-1 protein levels (Figure 5C). Interestingly, AM630 treatment does not produce any significant variation in the described experiments.

### 2.5. Effect of CB2 Receptor Stimulation on Macrophage Polarization

**Figure 6:**
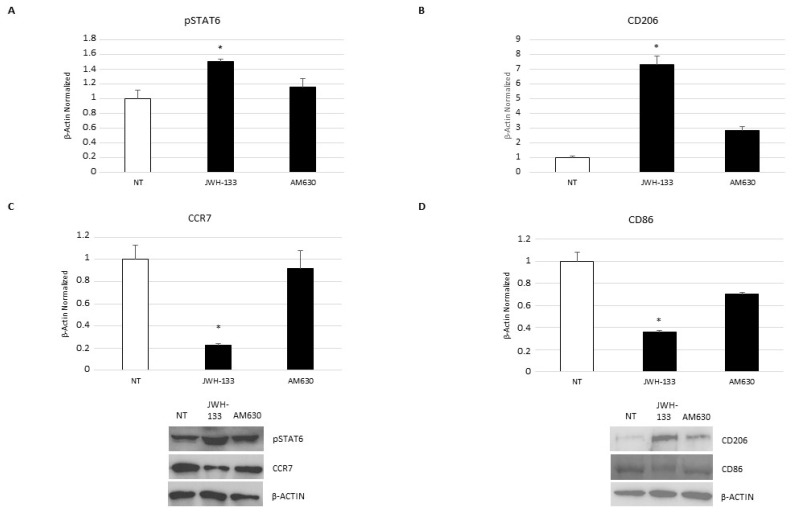
pSTAT6 (**A**), CD206 (**B**), CCR7 (**C**), and CD86 (**D**) protein expression levels in DMD-associated macrophages after 24-h treatment with JWH-133 and AM630, determined by Western blotting, starting from 15 μg of total lysates. The most representative images are displayed. The protein bands were detected through Image Lab. Ink software “BIORAD”, and the intensity ratios of immunoblots compared to non-treated (NT), taken as 1, were quantified after normalizing with respective controls. The relative quantification for these proteins’ expression, normalized for the housekeeping protein β-actin, is represented in the histogram as the mean ± SD. Students’ *t*-test has been used for statistical analysis. *, *p* ≤ 0.05 compared to NT (*p*-values: (**A**) 0.0541; (**B**) 0.0101; (**C**) 0.0066; (**D**) 0.0146).

With Western blotting, we studied the effect of treatments on M1 and M2 macrophages surface markers. JWH-133 exposure causes a significant increase in pSTAT6 and CD206 proteins, two of the most important M2 markers (Figure 6A,B), and significant reduction in CCR7 and CD86 protein expression level (Figure 6C,D), which are markers of the M1 phenotype. Interestingly, AM630 administration does not produce any significant variation.

## 3. Discussion

Macrophages are immune cells normally involved in several processes, namely, cell death, toxin clearance, wound healing, and others, acting as a “professional phagocytes” in both physiological and inflammatory/pathological conditions [46]. They are increasingly proposed as a therapeutic target in inflammatory and immune diseases [17] as well as in cancer [47,48]. The two prototypical macrophage phenotypes are M1, or classically activated macrophages with pro-inflammatory function, and M2, or alternatively activated macrophages with anti-inflammatory properties. They are generally balanced, but the ratio of M1/M2 is in favor of M1 subtypes in inflammatory and immune disease [17,18,48,49]. In the same manner, the switch of their phenotype towards the M2 one, namely, a reduction of the M1/M2 ratio, could apport benefits in different kind of diseases [17,18]. Since recent years, a greater interest has been evolved from the scientific communities in the role of the endocannabinoid system (ECS) in inflammatory diseases. CB2 receptor, a key element of ECS, is one of the main candidates in inflammatory diseases [25,50,51]. It is widely expressed on immune cells, including macrophages [19,28,52], and it is significantly de-regulated under inflammatory stimuli, thus letting hypothesize its involvement in the pathogenesis of several inflammatory and immune pathologies. In literature, it is reported that the selective stimulation of CB2—for example with JWH-133 selective agonist—inhibits the production of pro-inflammatory cytokines by macrophages, thus containing the exacerbation of inflammatory response [51]. To our knowledge, this is the first attempt to evaluate the possibility to target the CB2 receptor on macrophages’ primary cultures, obtained from DMD patients, to ameliorate their pro-inflammatory phenotypes that were causing the symptoms affecting these subjects. Currently, standard therapy for DMD patients is based on corticosteroids (CCs) administration, thus containing the inflammation and all related symptoms they experience. Despite the beneficial and therapeutic effects of CCS, they do not guarantee a complete restoration and are also responsible for a lot of severe long-term side effects [36]. Hence, it is necessary to set up a more efficient and less toxic long-term treatment. We firstly described the inflammatory state of these patients, focusing on prevailing macrophages phenotype and on their secretory activity by studying the released cytokine profile. In literature, it has been already reported that inflammation is a typical feature of DMD; in particular, high serum levels of TNF-α, IL-6, IL-7, and other pro-inflammatory molecules have been described [53]. In accordance, we observed an elevated release of pro-inflammatory cytokines IL-6 and IFN-γ by DMD-associated macrophages. Therefore, we confirm the already well-known alteration of cytokine levels in DMD patients, but we specifically related it to the macrophage M1 prevalence, providing a deeper cellular overview of this clinical evidence. Although IFN-γ is the principal cytokine exerting a protective role in immune response against microbial infections by activating macrophages [54], a constant and continuous hyperproduction of this molecular signal leads to a deleterious state of macrophage chronic activation, namely chronic inflammation. The high levels of CCR7 and CD86 proteins and the contemporary low levels in pSTAT6 and CD206 protein expression let us speculate that this inflammatory state is both a cause and an effect of a M1 phenotype prevalence [55,56]. Further evidence supporting this hypothesis comes from the observed high intracellular concentration of iron. Indeed, although M1 cells normally show high iron content that limits growth of pathogen agents and promotes inflammation, M2 macrophages are mainly responsible for iron release [48,57]. The observed high levels of intracellular iron do not only confirm the M1 macrophages prevalence, but they are also a further signal of inflammation. High levels of pro-inflammatory cytokines, mainly IL-6, as we observed, indeed cause iron retention in cells by the hepcidin pathway [29,34]. Inflammation is a self-sustaining event, given the interdependent and mutually stimulating relationship among the involved compounds. In our study, it emerged that DMD-associated macrophages are principally M1 macrophages and that they express low levels of the CB2 receptor. Our experimental results demonstrate that the proper and selective stimulation of the CB2 receptor could cause beneficial effects by rebalancing the levels of pro-inflammatory cytokines and reducing iron internalization. The reduction of IL-6, IFN-γ, and intracellular iron are clear evidence of the anti-inflammatory effect of CB2 stimulation. From our study, it emerged that JWH-133 treatment influenced iron internalization by modulating the expression of two of the main iron transporters: TfR1 and FPN-1. Indeed, although TfR1 decreased, with a consequent reduction in cell iron intake, FPN-1 increased, making more effective the iron export into extracellular environment. These results let us hypothesize that CB2 stimulation exerts its anti-inflammatory role in DMD in a double manner: by inhibiting pro-inflammatory cytokines further release and also by reducing iron internalization in macrophages. The modulation of iron metabolism appears directly related to CB2 effects on iron transporters, TfR1 and FPN-1, but it is also clearly mediated by the IL-6/hepcidin pathway. Beyond us, other authors also demonstrated that CB2 and TRPV1 receptor-stimulating molecules—for example cannabidiol (CBD), a phytocannabinoid extracted from *Cannabis sativa* L.,—reduce IL-6 release, thus triggering the hepcidin/FPN-1 cascade and exerting an anti-inflammatory role [58,59,60]. In the future, certainly, it could be interesting to unravel the depth of the underlying molecular mechanisms for the identified interactions. Being that IL-6 is an enhancer for M1 macrophages differentiation and function, its reduction contributes to the lowering of the existing M1 prevalence. Indeed, after stimulating macrophages with JWH-133, we observed an increase in pSTAT6 protein, which is a key molecule in the transition from M1 to M2 phenotype. As many authors already suggested, the phosphorylation of this protein regulates the M2 polarization, so it could be considered not only an M2 phenotype marker, but a marker of polarization progress toward the M2 type [55,56]. Our results highlight the promising possibility to target the CB2 receptor in DMD, leveraging anti-inflammatory effects mediated by macrophages, the most representative cells of the immune system, to improve short- and long-term outcomes in these patients.

## 4. Materials and Methods

### 4.1. Source of Macrophages

Macrophages were obtained from the peripheral blood of 10 DMD patients (median age 10 ± 2 years) and 10 healthy donors (median age 10.4 ± 2 years). Patients from both study groups were enrolled in the Department of Women, Child and General and Specialist Surgery of University of Campania “Luigi Vanvitelli”. Beside the high serum level of creatine kinase and transaminases, along with abnormal muscular function in male child, DMD diagnosis was confirmed by the DMD gene mutation [61].

### 4.2. Macrophages Primary Cultures

Macrophages were obtained from peripheral blood mononuclear cells (PBMCs). PBMCs were isolated by density gradient centrifugation (Ficoll 1.077 g/mL; Lympholyte, Cedarlane Laboratories Ltd., Uden, The Netherlands), diluted at 1 × 106 cells/mL in α-Minimal Essential Medium (α-MEM) (Lonza, Verviers, Belgium) supplemented with 10% fetal bovine serum (FBS) (Euroclone, Siziano, Italy), 100 IU/mL penicillin, and 100 g/mL streptomycin and L-glutamine (Gibco Limited, Uxbridge, UK), and plated in 24-well Cell Culture Multiwell. To obtain fully differentiated human macrophages, the PBMCs were cultured for 15 days in the presence of 25 ng/mL recombinant human macrophage colony-stimulating factor (rh-MCSF) (Peprotech, London, UK). Culture medium was replaced twice a week. Cells were cultured at 37 °C in a humidified atmosphere with 5% CO_2_. Macrophages derived from DMD patients were treated with JWH-133 and AM630 when they were completely differentiated. After 24 h of treatment exposure, cells were harvested for protein extraction, and cell cultures supernatants were collected to perform the iron assay and to analyze the release of several pro- and anti-inflammatory cytokines by means of enzyme-linked immunosorbent assay (ELISA).

### 4.3. Drugs and Treatments

JWH-133 and AM630 powder were purchased from Tocris (Avonmouth, UK) and were dissolved in PBS containing DMSO. DMSO final concentration on cultures was 0.01%. DMD-associated macrophages were treated with JWH-133 [100 nM] and AM630 [10 µM] for 24 h. Non-treated cultured macrophages were maintained in incubation media during the relative treatment time with or without vehicle (DMSO 0.01%).

### 4.4. Protein Isolation and Western Blotting

Proteins were extracted from treated and non-treated macrophages cultures using radio-immunoprecipitation assay (RIPA) Lysis Buffer (Millipore, Burlington, VT, USA) and following the manufacturer’s instructions. CB2, pSTAT6, CD206, CCR7, CD86, TfR1, and FPN-1 proteins were detected in total lysates from cell line cultures by Western blotting. Membranes were incubated overnight at 4 °C with these antibodies: anti-CB2 receptor antibody (1:250, Mouse. Santa Cruz Biotechnology, Dallas, TX, USA), anti-pSTAT6 antibody (1:500, Rabbit. Elabscience, Houston, TX, USA), anti-CD206 antibody (1:500, Mouse. Santa Cruz, TX, USA), anti-CCR7 antibody (1:500, Rabbit. Elabscience, Houston, TX, USA), anti-CD86 antibody (1:500, Rabbit. Elabscience, Houston, TX, USA), anti-TfR1 antibody (1:1000, Rabbit. Abcam, Cambridge, UK), anti-FPN-1 antibody (1:1000, Rabbit. Novus Biologicals, Italy). Reactive bands were detected by chemiluminescence (Immobilon Western Chemiluminescent HRP Substrate, Millipore, Burlington, MA, USA) on CHEMIDOC Bio-Rad (BioRad, Hercules, CA, USA). A mouse monoclonal anti β-actin antibody (1:5000, Elabscience, Houston, TX, USA) was used to check for comparable protein loading and as a housekeeping protein. Images were captured, stored, and analyzed using Image Lab. Ink software.

### 4.5. ELISA

Several ELISA assays were performed in order to determine IL-6, IL-4, IL-10, and IFN-γ concentration in cell cultures supernatants by using commercially available Human ELISA Kits (Invitrogen by Thermo Fisher, Waltham, MA, USA) according to the manufacturer’s instructions. Briefly, the microplates were coated with monoclonal antibodies specific to the cytokines. Standards and supernatants were pipetted into the wells of the microplate and were run in duplicate. After the plate was washed, enzyme-linked polyclonal antibodies specific for IL-4, IL-10, IL-6, and IFN-γ were added to the wells. The reaction was revealed by the addition of the substrate solution. The optical density was measured at a wavelength of 450 nm by using the DAS Italy plate reader (DAS Italy, Palombara Sabina—Italia). Cytokines concentrations (pg/mL) were determined against a standard concentration curve.

### 4.6. Iron Assay

After 24-h exposure to JWH-133 and AM630, cell culture supernatants were collected to measure iron (III). The assay was performed by using the Iron Assay Kit (Abcam, Cambridge, UK) according to the manufacturer’s instructions. Briefly, standards and macrophages supernatant were pipetted into the wells and were incubated with an acidic buffer to allow iron release. Then, an iron probe at 25 °C for 60 min was added, protected from light. Released iron reacted with the chromogen resulting in a colorimetric (593 nm) product, proportional to the iron amount. The optical density was measured at a wavelength of 593 nm by using the DAS Italy plate reader (DAS Italy, Palombara Sabina—Italia.). Iron (II) and Total Iron (II + III) contents of the test samples (nmol/μL) were determined against a standard concentration curve. Iron (III) content can be calculated as: Iron (III) = Total Iron (II + III) − Iron (II).

### 4.7. Statistical Analysis

Statistical analyses on all the performed experiments were executed by using the Students’ *t* test to evaluate differences between quantitative variables. Each experiment was performed on samples deriving from three different subjects and data expressed as the mean ± SD. A *p* value ≤ 0.05 was considered statistically significant.

## 5. Conclusions

In conclusion, we discussed for the first time the inflammatory state of DMD-associated human macrophages in primary cultures and, overall, the involvement of CB2 receptor in the pathogenesis of the typical chronic inflammation affecting these subjects. Our results suggest the possibility to target the CB2 receptor with beneficial therapeutical effects, limiting the release of further pro-inflammatory cytokines and enhancing the differentiation of anti-inflammatory M2 macrophages.

In future, it will be of great interest to test the effect of CB2 agonist-CCs combined use in DMD-associated macrophages. In 2019 we demonstrated that in mesenchymal stromal cells (MSCs) from immune thrombocytopenia (ITP) patients, the co-administration of Dexamethasone—the glucocorticoid most frequently used in ITP patients—with JWH-133 resulted in an extremely potentiated anti-inflammatory effect [25]. This synergism could represent a cardinal element in DMD therapy as well, allowing to reduce CCs dosage and consequently the related side effects. Certainly, further in vitro and in vivo studies are needed to validate and translate our findings into a path to clinically practice in order to improve the therapeutic efficacy for DMD patients.

## Data Availability

Not applicable.
